# Vagal Afferent Processing by the Paratrigeminal Nucleus

**DOI:** 10.3389/fphys.2019.01110

**Published:** 2019-08-28

**Authors:** Alexandria K. Driessen

**Affiliations:** School of Biomedical Science, Department of Anatomy and Neuroscience, University of Melbourne, Parkville, VIC, Australia

**Keywords:** respiratory, nociception, jugular ganglia, paratrigeminal connectome, cough

## Abstract

The paratrigeminal nucleus is an obscure region in the dorsal lateral medulla, which has been best characterized as a collection of interstitial cells located in the dorsal tip of the spinal trigeminal tract. The paratrigeminal nucleus receives afferent input from the vagus, trigeminal, spinal, and glossopharyngeal nerves, which contribute to its long-known roles in the baroreceptor reflex and nociceptive processing. More recently, studies have shown that this region is also involved in the processing of airway-derived sensory information. Notably, these studies highlight an underappreciated complexity in the neuronal content and circuit connectivity of the paratrigeminal nucleus. However, much remains to be understood about how paratrigeminal processing of vagal afferents is altered in disease. The aim of the present review is to provide an update of the current understanding of vagal afferent processing in the paratrigeminal nucleus and to explore how dysregulation at this site may contribute to vagal sensory neural dysfunction during disease.

## Introduction

Vagal afferents innervating visceral tissues are well described to terminate in the medullary nucleus of the solitary tract (Cajal, 1909; Donoghue et al., 1982; Kubin et al., 1985; Mutoh et al., 2000; McGovern et al., 2012). However, it has also long been known that there are additional brainstem termination sites for vagal afferent fibers in the dorsal lateral medulla, including a region known as the paratrigeminal nucleus (Cajal, 1909; Altschuler et al., 1989; McGovern et al., 2012). Recent studies using viral circuit tracing of the airway sensory nervous system demonstrated that only a specific subset of vagal afferents project to the paratrigeminal nucleus (McGovern et al., 2015a) and since this observation there has been significant progress made toward understanding the role of the paratrigeminal nucleus in airway defense (Driessen et al., 2015, 2018). This brief review aims to bring together anatomical and functional investigations of the paratrigeminal nucleus to present a case for why this nucleus should be acknowledged as a second key processing site for vagal afferent inputs to the central nervous system.

## Neuroanatomical Studies of the Paratrigeminal Nucleus and its Connectome

### Anatomically Defining the Paratrigeminal Nucleus

The paratrigeminal nucleus is an obscure medullary region that was first characterized in the 1960s by Albert Rhoton. He defined this nucleus, in the monkey, as a collection of interstitial neurons located within the dorsal segment of the spinal trigeminal tract that is located in the lateral aspect of the medulla (Rhoton et al., 1966). Neurons in this region receive a diverse range of sensory inputs arising from trigeminal, glossopharyngeal, vagal sensory, and upper cervical dorsal root sensory ganglia (Ciriello et al., 1981; Panneton and Burton, 1981; Nomura and Mizuno, 1984; Altschuler et al., 1989; McGovern et al., 2012; Mahadi et al., 2019). Subsequent studies have identified comparable interstitial cells in the rat, guinea pig, cat, and human (Chan-Palay, 1978; Nasution and Shigenaga, 1987; Phelan and Falls, 1989; Pinto et al., 2006; Driessen et al., 2015; McGovern et al., 2015a). Phelan and Falls (1989) were the first to investigate the rostro-caudal extent of the paratrigeminal nucleus in the rat, showing that presumptive paratrigeminal neurons extend rostrally for approximately 1.5–2 mm from caudal to obex. Similarly, in the guinea pig, the paratrigeminal nucleus spreads 1.5 mm with the highest density of neurons located in the caudal (−0.4 mm to obex) and central (at the level of obex) aspects of this nucleus (Driessen et al., 2018).

Investigations of the cellular composition of the paratrigeminal nucleus have shown it to contain morphologically distinct subpopulations of neurons, as well as pronounced numbers of glia and astrocytes in its most superficial layers (Chan-Palay, 1978; Phelan and Falls, 1989; Saxon and Hopkins, 2006). The paratrigeminal nucleus is also enriched with a peptidergic neuropil, predominately expressing the neuropeptides calcitonin gene-related peptide and substance P that presumably represent the central terminal processes of the sensory neural inputs (Saxon and Hopkins, 2006; Caous et al., 2012; Driessen et al., 2015). Recent immunohistochemical characterization of the paratrigeminal nucleus in the guinea pig has demonstrated at least two distinct paratrigeminal neuron subtypes based on their immunoreactivity for either calbindin or the neurokinin 1 receptor (Driessen et al., 2018), analogous with the dorsal horn nociceptive system that contains phenotypically comparable neurons in receipt of nociceptive inputs (Li et al., 1999). However, this is unlikely a complete representation of the neuron subtypes that exist in the paratrigeminal nucleus and further neuronal heterogeneity may exist, like that in the dorsal horn (Hu et al., 2016; Li et al., 2016; Jurcakova et al., 2018). It seems reasonable to speculate that this under-appreciated complexity of the paratrigeminal nucleus likely confers functional significance with respect to the integration and processing capabilities of this nucleus.

### Vagal Afferent Terminations in the Paratrigeminal Nucleus

Vagal afferents have their cell bodies located in either the jugular (superior) or nodose (inferior) vagal ganglia (Canning et al., 2004; Undem et al., 2004; McGovern et al., 2012). These two ganglia are derived from different embryological origins (D’Autréaux et al., 2011; Nomaksteinsky et al., 2013) and this confers significant differences in the physiology, pharmacology, and neuroanatomy of the neurons originating in each (Kwong et al., 2008; Chang et al., 2015; McGovern et al., 2015a,b; Nonomura et al., 2017; Kupari et al., 2019). Studies of airway projecting vagal sensory neurons in the mouse and guinea pig indicate that the visceral placodal nodose-derived neurons are characteristically non-peptidergic consisting of myelinated Aδ- or Aβ- fibers and unmyelinated C-fibers (Riccio et al., 1996; Undem et al., 2004; Mazzone and Undem, 2016) that can be selectively activated by serotonin 5-HT3 receptor agonists and adenosine 5′-triphosphate (ATP) as they uniquely express the purinergic receptors P2X2 and P2X3 (Kwong et al., 2008; Potenzieri et al., 2012). On the other hand, airway somatic neural crest-derived jugular neurons are classically characterized as non-peptidergic Aδ-fibers or peptidergic C-fibers unresponsive to both 5-HT and ATP due to lack of expression of 5HT3 and P2X2 receptors (Riccio et al., 1996; Canning et al., 2004; Undem et al., 2004; Potenzieri et al., 2012). Recent single-cell RNA sequencing of unidentified nodose and jugular afferent neurons in the mouse reveal comparable phenotypic differences, with jugular neurons demonstrating more similarities to neurons of the dorsal root ganglia, which is underpinned by their developmental origin (Kupari et al., 2019).

**Figure 1:**
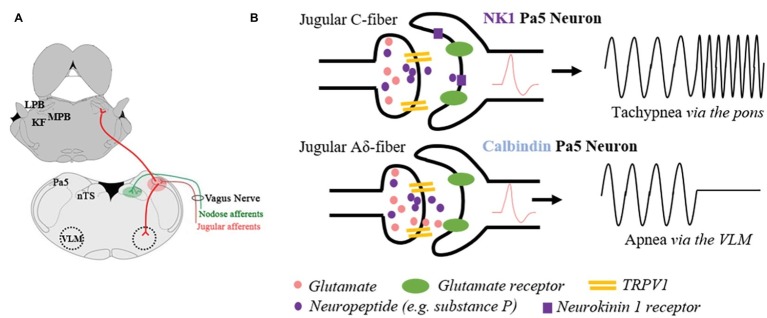
Schematic representation of the anatomical and functional specificity of the paratrigeminal nucleus (Pa5). **(A)** Illustration of jugular vagal afferents (red) specifically terminating in the Pa5, located in the dorsal-lateral medulla. Conversely, the nodose vagal afferents (green) exclusively project to the nucleus of the solitary tract (nTS). Jugular vagal afferent inputs are first synaptically integrated with Pa5 neurons, which differentially project to additional bulbar nuclei involved in cardiorespiratory control. Depicted are a subset of Pa5 neurons projecting to the ventrolateral medulla (VLM), and a separate population ascending to the pontine nuclei that collectively include the Kölliker Fuse (KF) nucleus and lateral (LPB) and medial (MPB) parabrachial nuclei. **(B)** Stylized diagram depicting a hypothesis for how the neuroanatomical organization of the Pa5 underpins functional specificity at this site based on studies of laryngeal-evoked reflexes in guinea pigs (Driessen et al., 2018). Non-peptidergic jugular A-fibers expressing the transient receptor potential vanilloid 1 (TRPV1) appear to mediate the abrupt cessation in breathing (apnea) observed when the larynx is electrically or chemically stimulated. Synaptically, this response is dependent on fast ionotropic glutamatergic neurotransmission in the Pa5, and it is hypothesized that calbindin expressing Pa5 neurons projecting to cardiorespiratory cells of the ventrolateral medulla are principally involved. Peptidergic (e.g., substance P containing fibers) inputs to the Pa5 are likely responsible for increased respiratory drive (i.e., tachypnea). Physiological or pathophysiological conditions that allow for neuropeptide release will activate NK1 receptor expressing Pa5 neurons, which possibly ascend the neuraxis and terminate diffusely in the pons. This pathway may contribute to respiratory and autonomic processing *via* these pontine circuits or excite bulbar-circuits involved in sensory discrimination.

In the guinea pig, viral tract tracing using an adeno-associated virus encoding green fluorescent protein (AAV2/8-eGFP) microinjected into the vagal sensory ganglia revealed that vagal afferents predictably project to the nucleus of the solitary tract, and additionally to the caudal and central regions of the paratrigeminal nucleus, which are the medullary levels that align with the highest density of postsynaptic neurons (Driessen et al., 2018). Similarly, tracing in the rat of vagally innervated viscera, the pharynx and larynx show bilateral afferent projections to both the nucleus of the solitary tract and the paratrigeminal nucleus (Saxon and Hopkins, 2006). Furthermore, direct electrical stimulation of the vagus nerve (Rutherfurd et al., 1992) or larynx (Wang et al., 2015) results in neuronal activation of paratrigeminal neurons. These data raise the question of whether paratrigeminal projecting vagal afferents represent a distinct population of neurons from the nodose or jugular vagal ganglia. McGovern et al. (2015b) addressed this by performing tracing studies of the afferents innervating different levels of the respiratory tree. In doing so, they showed that the lungs are predominately innervated by afferents from the nodose ganglia, while the upper airways (larynx, trachea, and mainstem bronchi) are innervated by the neurons derived from the jugular ganglia (McGovern et al., 2015b). Subsequent dual retrograde neural tracing studies in rats and guinea pigs confirmed the existence of differential vagal inputs to brainstem processing sites demonstrating that nodose vagal afferents project exclusively to the nucleus of the solitary tract, while the jugular vagal ganglia afferents project almost entirely to the paratrigeminal nucleus (Figure 1A; Driessen et al., 2015; McGovern et al., 2015a). These studies suggest that two parallel vagal sensory circuits exist, broadly classified as either a nodose-nucleus of the solitary tract processing pathway or jugular-paratrigeminal processing pathway.

### Paratrigeminal Nucleus Output Connectivity

Neuronal tracing studies in both rat and guinea pig have shown output projections from paratrigeminal neurons to bulbar nuclei integral to autonomic and nociceptive processing, including the nucleus of the solitary tract, spinal trigeminal nuclei, rostral ventrolateral medulla, nucleus ambiguus, reticular nuclei, parabrachial nuclei, Kölliker-Fuse nucleus, and principal sensory trigeminal nucleus (Feil and Herbert, 1995; Saxon and Hopkins, 1998; Caous et al., 2001; de Sousa Buck et al., 2001; Pinto et al., 2006; McGovern et al., 2015b; Driessen et al., 2018). While no monosynaptic projections were observed above the level of the pons in the guinea pig (Driessen et al., 2018), ascending projections directly to the somatosensory thalamus (ventroposterior medial thalamus) have been observed in rats (Saxon and Hopkins, 1998; Pinto et al., 2006; McGovern et al., 2015a). Interestingly, dual cholera-toxin retrograde tracing studies have shown different projection patterns of subpopulations of paratrigeminal neurons (Saxon and Hopkins, 1998; Pinto et al., 2006; Driessen et al., 2018). For example, Pinto et al. (2006) showed topography of pontine projections could be mapped across the rostro-caudal extent of the paratrigeminal nucleus, indicative of selective pontomedullary connectivity. Additionally, in the rat, separate paratrigeminal neurons project to the parabrachial nuclei compared to the ventroposterior medial thalamus (Saxon and Hopkins, 1998), while in the guinea pig there are distinct neurons that project to the parabrachial nuclei compared to the ventrolateral medulla (Figure 1A; Driessen et al., 2018). Given that at least two populations of phenotypically different paratrigeminal neurons exist (expressing neurokinin 1 receptors or calbindin; Driessen et al., 2018), it will be important to investigate whether these populations preserve a level of connectivity specificity.

## The Physiological Role of the Paratrigeminal Nucleus in Vagal Afferent Regulation of Cardiorespiratory Control

Vagal afferent inputs from aortic baroreceptors to the brainstem play an essential role in the regulation of blood pressure *via* circuits involving the ventrolateral medulla (Bonham and Jeske, 1989; Ruggiero et al., 1996; Choong et al., 2013; Koganezawa and Paton, 2014; Tallapragada et al., 2016; Ghali, 2017). Although many baroreceptive afferents terminate centrally in the nucleus of the solitary tract, neurons in the paratrigeminal nucleus also demonstrate baroreceptor-related activity (Caous et al., 2001; de Sousa Buck et al., 2001). In the rat, up to 72% of paratrigeminal neurons are barosensitive and of these almost all show rhythmic activity with the cardiac cycle (Balan Júnior et al., 2004; Sousa and Lindsey, 2009). Importantly, baroreceptor reflex evoked changes in cardiovascular activity is modulated by altering synaptic processing in the paratrigeminal nucleus. Thus, microinjection of bradykinin into the paratrigeminal nucleus results in increased arterial pressure, while lesioning this nucleus increases resting arterial pressure and heart rate and impairs baroreceptor reflex sensitivity (Lindsey et al., 1997; Sousa and Lindsey, 2009). Although not proven, these effects are presumably mediated by paratrigeminal neuronal connectivity with the ventrolateral medulla. However, some entrainment of barosensitive neural activity in the paratrigeminal nucleus and the nucleus of the solitary tract suggests functional interconnectivity between these vagal sensory processing sites (Balan Júnior et al., 2004).

Vagal afferents are essential for maintaining appropriate airway patency and adequate respiration. In anesthetized animals, nodose vagal stimulation results in apnea *via* slowly adapting lung stretch receptors (Knowlton and Larrabee, 1946; Backman et al., 1984; Kubin et al., 1985; Widdicombe, 2001; Nonomura et al., 2017), tachypnea *via* rapidly adapting mechanoreceptors or C-fibers (Chou et al., 2008), or cough *via* the activation of the specialized mechanically sensitive nodose Aδ-fibers (the cough receptor) in the upper airways (Canning et al., 2004, 2006; Mazzone et al., 2009). A full description of nodose-evoked responses is beyond the scope of this review.

Conversely, only a few studies have specifically investigated the effects of jugular vagal stimulation on respiration. In guinea pigs, jugular vagal afferents evoke an apnea in anesthetized animals when stimulated and mediate coughing in response to inhaled noxious chemicals in conscious animals (Chou et al., 2008, 2018; Driessen et al., 2015, 2018). The apneic response, readily induced from the guinea pig larynx (Driessen et al., 2015, 2018), is perhaps consistent with the reported role of the paratrigeminal nucleus in the diving reflex whereby stimulation of trigeminal afferents in the nose promotes breath holding (Panneton et al., 2000; McCulloch et al., 2018). Indeed, the jugular vagal innervation to the larynx is particularly dense an either electrical stimulation or topically applied capsaicin evokes a profound withdrawal of respiratory drive. This reflex response to laryngeal stimulation is abolished by generalized inhibition of the paratrigeminal nucleus, but not the nucleus of the solitary tract, using microinjections of the GABA receptor antagonist muscimol (Driessen et al., 2015). Furthermore, laryngeal-evoked reductions in breathing are predominately dependent upon glutamatergic neurotransmission in the paratrigeminal nucleus, while neuropeptides appear to play a neuromodulatory role (Figure 1B; Driessen et al., 2018). Consistent with this, apneas can also be induced by activation of neurons in the paratrigeminal nucleus by direct microinjection of glutamate (Driessen et al., 2018). Together, these functional data support the role of jugular vagal afferents in suppressing respiratory drive.

Intriguingly, when the central terminals of afferents in the paratrigeminal nucleus are activated using microinjection of capsaicin, a slowly developing but long-lasting tachypnea is observed, distinctly opposite to the abrupt apneas described above. This tachypneic response is unaffected by glutamate antagonism but abolished in the presence of substance P receptor antagonists (Driessen et al., 2018). Whether this effect is mediated by vagal afferent nerve fibers is not known. However, these data suggest that at least two distinct neural circuits arise from the paratrigeminal nucleus and display opposing effects on respiration.

The potential role of the paratrigeminal nucleus in cough has not been investigated but is predicted based on the known role of jugular vagal afferents in evoking coughing (Hewitt et al., 2016; Chou et al., 2018). In conscious guinea pigs, inhaled chemical stimuli that activate both nodose and jugular nociceptors (e.g., capsaicin, bradykinin, citric acid) reliably evoke cough, whereas selective stimuli of nodose nociceptors (ATP, adenosine and 5HT) do not evoke coughing (Chou et al., 2018). Furthermore, nicotine selective for jugular afferents (Gu et al., 2008; Tao et al., 2019) readily induces cough (Forsberg et al., 1988; Lee et al., 2007), as does Arnold’s reflex involving stimulation of the auricular branch of the vagus nerve (Ryan et al., 2014; Dicpinigaitis et al., 2019) whose fibers are derived from the jugular vagal ganglia (DuBois and Folley, 1937). A hypersensitive Arnold’s reflex has now been shown to be a biomarker that defines chronic cough (Dicpinigaitis et al., 2019) and in turn could be a promising therapeutic target given that transcutaneous vagal stimulation at this site has been shown to be important for a variety of cardiovascular conditions (Murrary et al., 2016). How jugular afferent inputs are integrated in the paratrigeminal nucleus to coordinate cough and other changes to respiration has not been studied.

## The Paratrigeminal Nucleus and Conscious Perception of Vagal Afferents

The ascending circuits from the paratrigeminal nucleus largely involve somatosensory processing regions of the thalamus and cortex (Ralston, 2005; McGovern et al., 2015a) and this may implicate the jugular-paratrigeminal pathway in the conscious perception of jugular vagal inputs to the brain. Indeed, the paratrigeminal nucleus has been shown to be involved in processing pain (Ma et al., 2005; Koepp et al., 2006). With respect to the vagal system, this has been best studied for airway jugular nociceptors. In guinea pigs and rats, jugular vagal afferents are most concentrated in the large proximal airways (McGovern et al., 2015b; Driessen et al., 2018), which are the structures most commonly associated with perceivable sensations (Ando et al., 2014; Hilton et al., 2015). In this regard, it is interesting that previous studies have shown that jugular vagal afferents are important for the induction of coughing evoked by noxious chemical stimuli in conscious but not anesthetized animals (Mazzone et al., 2005), where conscious perception is presumably absent. In humans, stimulation of upper airway structures innervated by jugular afferents evokes a sensory experience known as the urge-to-cough (Mazzone et al., 2011) that while in healthy individuals is relieved by coughing; in disease, these irritations persist contributing to chronic cough and increased morbidity associated with respiratory disease (Chung, 2011; Morice, 2013). A hypothesis is that the urge-to-cough and associated cough is dependent upon jugular C-fibers synapsing with neurokinin 1 receptor expressing paratrigeminal neurons. In the medullary dorsal horn, neurokinin 1 receptor expressing neurons are excitatory and project to higher order circuits that are important for discriminative and emotive processing associated with orofacial pain (Cameron et al., 2015; Li et al., 2017). Furthermore, with respect to cough, neurokinin 1 receptor antagonists have shown to be antitussive in both animal and human studies (Amadesi et al., 2001; Chapman et al., 2004; El-Hashim et al., 2004; Grobman and Reinero, 2016). Therefore, future studies aiming to define the organization of the jugular-neurokinin 1 receptor paratrigeminal circuitry in cough will improve our understanding of the airway sensory nervous system and in turn generate new and improved treatment options for sufferers of respiratory disease.

## Conclusion: Significance and Future Directions

The paratrigeminal nucleus has an undeniable role in the processing of vagal afferent inputs. It has roles in both autonomic and nociceptive processing acting akin to the nucleus of the solitary tract and dorsal horn respectively in a context specific manner. However, the extent of this involvement has largely remained unstudied, perhaps reflecting the technical difficulties associated with studying jugular vagal afferents and this small region of the brainstem. Consequently, many questions remain about the mechanistic processing of different sensory modalities and their integration in the paratrigeminal nucleus. To this end, it will be interesting to determine the relative contributions of jugular A- and C-fiber sensory neural inputs into different paratrigeminal circuits (Figure 1B) and to define the physiological circumstance under which neuropeptides are released. Indeed recent advancements in molecular phenotyping of vagal sensory neurons (Kupari et al., 2019), the development of new viral vector systems and advances in imaging technologies may lead to novel approaches for investigating this neuronal circuitry.

However, it is also important to recognize that our current understanding of this topic is derived predominately from studies in healthy animals. Therefore, we likely have an under-appreciation of the importance of this nucleus in aberrant vagal afferent processing, which is well known to accompany the symptoms of disease. As such, it will be intriguing to investigate the potential plasticity in afferent processing that occurs in the paratrigeminal nucleus in a disease context, as this may provide new insights into the manifestation of heightened sensitivity and exaggerated reflexes that are characteristic of many visceral conditions. The trigeminal system (including the paratrigeminal area) has been implicated as a key region in chronic pain arising from the face (Imbe and Ren, 1999; Yamazaki et al., 2008). Under these conditions, the neurons of the trigeminal sensory nuclei have increased excitability that is likely underpinned by increased neuropeptide production and altered neuron-glial cross talk (Devesa et al., 2014; Romero-Reyes et al., 2015; Yang et al., 2016; Chen et al., 2017). In addition, changes in higher order circuitry have been described (Hayes et al., 2017). Similar sensory plasticity in the paratrigeminal nucleus may occur in visceral diseases and this could be especially relevant in conditions of vagal hypersensitivities, in which altered neurochemistry of jugular-paratrigeminal afferent processing may be an underlying feature of the associated sensory disturbances. This should be a matter of interest because sensory hypersensitivities accompanying visceral disease have proven difficult to treat and the paratrigeminal nucleus may represent a novel alternate target for therapeutic intervention.

## Author Contributions

The author confirms being the sole contributor of this work and has approved it for publication.

### Conflict of Interest Statement

The author declares that the research was conducted in the absence of any commercial or financial relationships that could be construed as a potential conflict of interest.
